# A case report for a diagnostic dilemma of a giant intra-abdominal cyst with an uncertain origin

**DOI:** 10.1016/j.ijscr.2020.01.036

**Published:** 2020-02-06

**Authors:** Nern Hoong Kao

**Affiliations:** Department of General Surgery, Changi General Hospital, Singapore

**Keywords:** Giant cyst, Intra-abdominal cyst

## Abstract

**Introduction:**

Giant intra-abdominal cystic lesions are seldom encountered and can post a diagnostic challenge pre-operatively. These often present as increasing abdominal size and from its mass effect.

**Presentation of case:**

Here, we present a case of a 58 year-old gentleman with worsening bloating and abdominal distension. A contrasted CT scan of the abdomen revealed a giant intra-abdominal cyst with no definite organ of origin. He underwent a laparotomy and excision of the giant cyst which was not found to be attached to any organ or mesentery. This resulted in resolution of his symptoms and a drastic improvement in his appetite.

**Discussion:**

It is often difficult to identify the origin of giant intra-abdominal cysts as pre-operative imaging may show it abutting multiple organs due to its size. Common intra-abdominal cysts include mesenteric, ovarian or peritoneal cysts. A precipitating history such as pancreatitis or surgical implants can suggest pseudocysts. Surgical excision alone is curative but can be difficult due to the size. Controlled intra-operative aspiration can aid in visualization and dissection.

**Conclusion:**

Giant intra-abdominal cystic lesions cause significant discomfort due to its mass effect. Pre-operative investigations may not identify its cause. Surgical excision is recommended to diagnostic and therapeutic purposes.

## Highlights

• Large intra-abdominal cyst identified on contrast CT scan of the abdomen and pelvis.
• No organ of origin was identified on the contrast CT scan.
• The cyst was not found to be originating from the mesentery or an organ.
• Controlled decompression helps with retraction and visualization for dissection.
• Surgical excision resulted in complete resolution of the patient’s symptoms.

## 1 Background

Giant intra-abdominal cysts (GIC) are seldom encountered and even more so in the adult population. This is due to the availability of radiological imaging that allow for an earlier diagnosis at a smaller size. Intra-abdominal cysts may remain asymptomatic until they achieve a certain size of which the patient may encounter non-specific complaints such as bloating and abdominal distension. The majority of abdominal pseudocysts occur as a result of a ventriculoperitoneal shunt or as sequelae of pancreatitis. Here, we present a case of a giant intra-abdominal pseudocyst with no risk factors or contributory cause and which represented a diagnostic dilemma. The case is reported in line with the SCARE criteria [1].

## 2 Case presentation

**Fig. 1:**
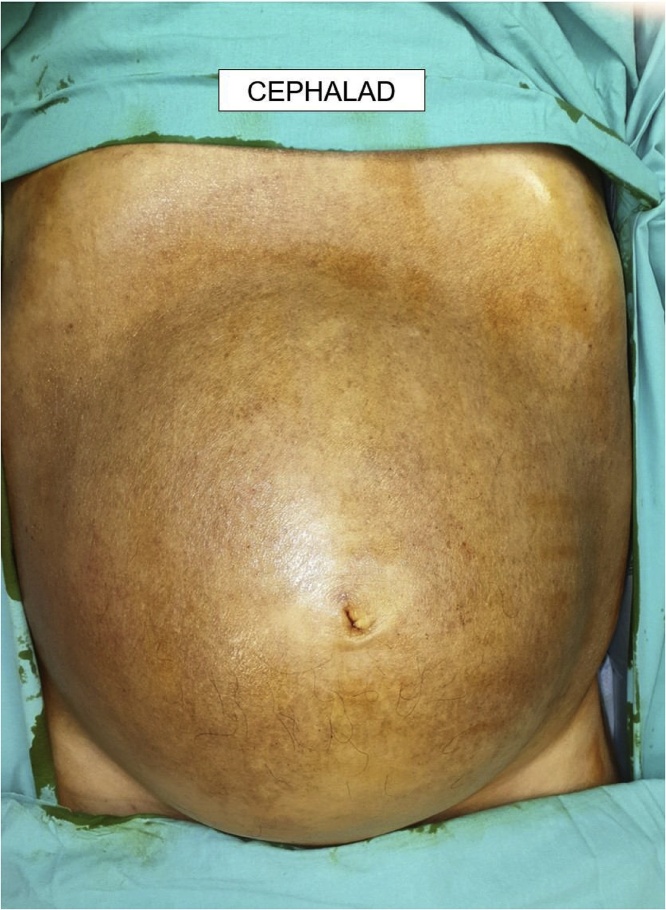
Anterior view of the patient’s abdomen showing distension from the intra-abdominal cyst.

**Fig. 2:**
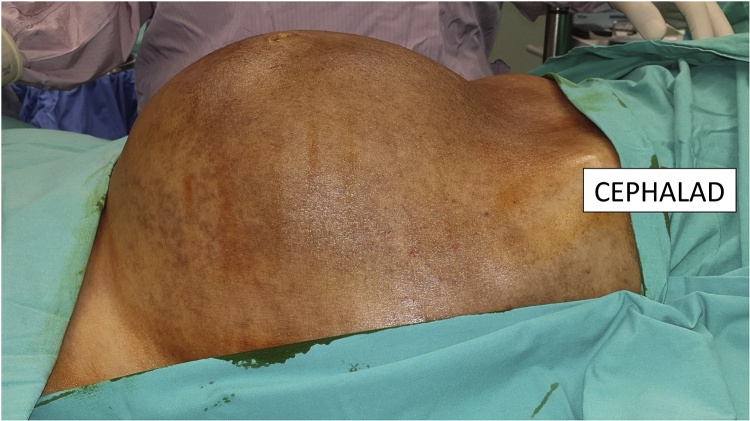
Left lateral view of the patient’s abdomen showing distension from the intra-abdominal cyst.

**Fig. 3:**
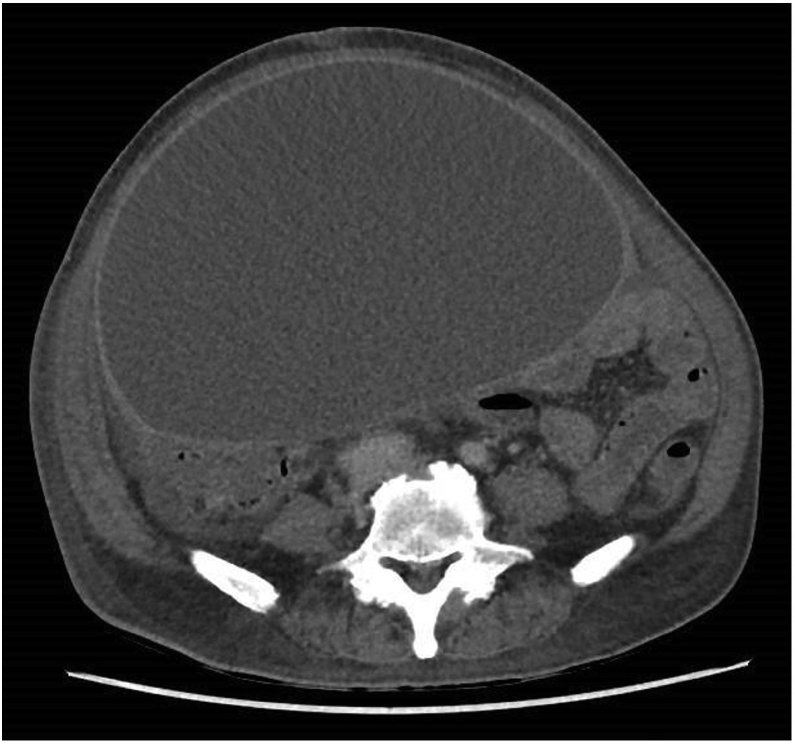
Contrast CT (axial view) showing large well-circumscribed homogenous hypoechoic cystic lesion with mass effect.

**Fig. 4:**
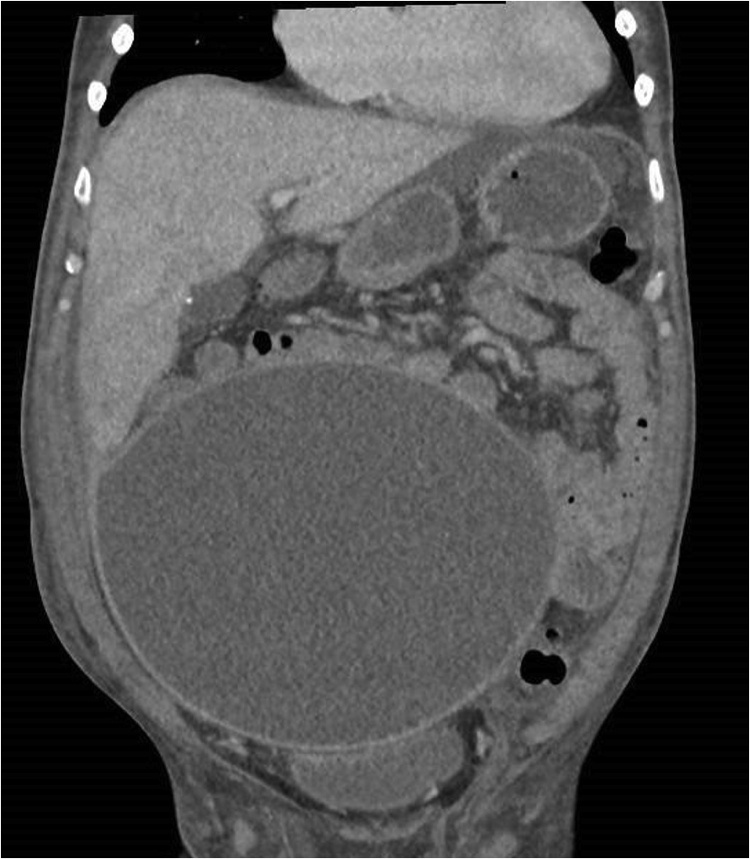
Contrast CT (coronal view) showing same lesion with a uniform wall and no abnormally thickened portion.

**Fig. 5:**
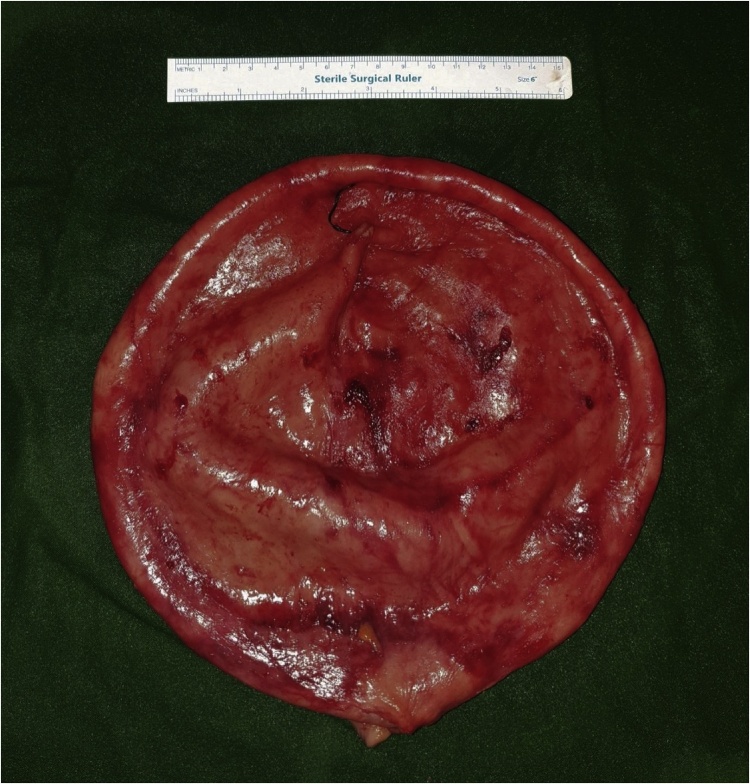
Decompressed thick-walled cyst excised in its entirety. Suture marks site of intra-operative decompression.

**Fig. 6:**
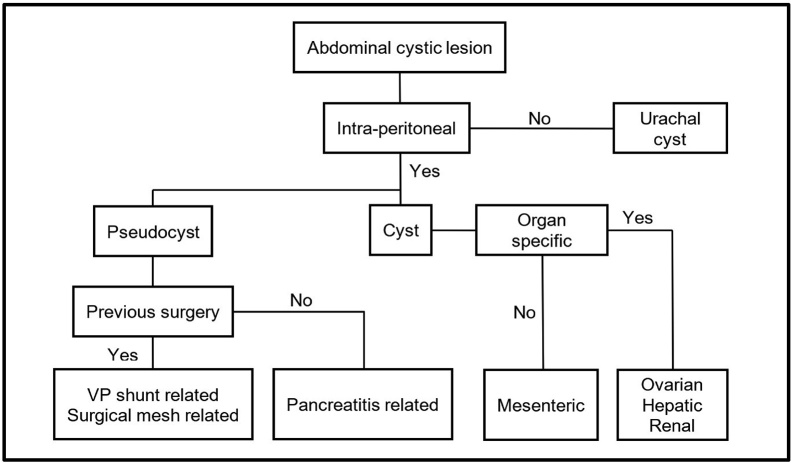
Flowchart depicting possible differentials of a giant abdominal cystic lesion.

A 58 year-old gentleman presented to the hospital with worsening bloating and a gradual increase in his abdominal girth. He had also noted a loss of weight of more than 10 kg over the last 2 years. The patient otherwise denied any abdominal pain or change in his bowel habit. He was known to have a history of well-controlled diabetes mellitus, hypertension, hyperlipidaemia and atrial fibrillation. There was no previous history of pancreatitis or abdominal surgery. The patient had recently undergone a gastroscopy and colonoscopy the previous year for iron deficiency anaemia. This had shown gastritis as well as the presence of pandiverticular disease and a sub-centimeter colonic polyp. Histology showed the polyp to be a tubular adenoma with low-grade dysplasia (Fig. 1, Fig. 2, Fig. 3, Fig. 4, Fig. 5, Fig. 6).

Clinical examination showed an adequately nourished gentleman but with a large abdominal mass occupying most of his abdomen. It was possible to feel over the superior edge but the inferior edge extended into the pelvis. The mass was non-tender on palpation. Digital rectal examination was unremarkable.

In view of the above findings, the patient underwent a computed tomography (CT) of the abdomen and pelvis. This showed a large 25 × 17 × 22 cm cystic lesion extending from the mid-abdomen to the pelvis. The lesion was thin walled and contained homogenous low density fluid (14 Hounsfield unit). There was no septations, irregularity or abnormal thickening of the cyst wall. The cyst was noted to have a mass effect but not invading the surrounding bowel loops and the urinary bladder. It was found to be separate from the liver and the kidneys. The pancreas was normal in appearance. The CT scan was otherwise unable to identify the origin of the giant cyst.

As this was a thin walled cyst with no irregular or solid component, a fine needle aspiration (FNA) was not suitable as there was no specific area to target. Aspiration of the fluid was also unlikely to yield any meaningful finding for diagnosis. Further imaging such as a MRI would also not help in identifying the origin or the diagnosis of the cyst. The possibility of a mesenteric or omental cyst was therefore discussed and surgical excision was offered to the patient. Tumour markers were not performed as it would not have affected the management.

An elective exploratory laparotomy was performed via a midline incision. A giant cyst was immediately identified but was found to have multiple adhesions to the peritoneum, omentum, mesentery, urinary bladder as well as the small and large intestines. However, the cyst was not found to be originating from any of the above organs or the vas deferens. The cyst was entirely within the abdominal cavity and did not originate from within the mesentery. A controlled decompression of the cyst was made through a purse-string encircled incision in the anterior wall. This was performed to aid with retraction and visualization of the posterior surface. Thick purulent fluid was aspirated until dry. The cyst was subsequently excised in whole.

The patient underwent an uneventful recovery with a brief period of expected post-operative ileus. He was discharged on post-operative day 6. Follow-up visits showed complete resolution of his initial symptoms and a vast improvement in his appetite.

Culture of the fluid was positive for *Streptococcus agalactiae*. Fungal culture and tuberculosis polymerase chain reaction (TB PCR) tests were negative. Cytology of the fluid showed mainly neutrophils.

Histological examination of the cyst showed thick fibrous walls covered with coarse fibrillary strands admixed with fibrin. There were also large numbers of mature IgG plasma cells with aggregates of neutrophils and scattered lymphocytes. No viable epithelial lining was identified. The walls stained positive for AE1/3 suggesting myofibroblasts. They were negative for CD117 and DOG-1 and therefore not suggestive of a gastrointestinal stromal tumour.

This was therefore treated as a benign cyst of an undetermined origin.

The patient was last seen in clinic two months after his surgery with a significant improvement in his appetite and oral intake. He was thereafter discharged from follow-up as the likelihood of recurrence was low considering that the cyst had been excised entirely with no remnant wall left behind.

## 3 Discussion

GIC are seldom encountered in the recent times as they are often picked up at a smaller size due to the ease of medical access and imaging. Patients tend to present with non-specific symptoms such as abdominal fullness, distension, change in bowel habit, pain or nausea and vomiting [2,3]. These occur as a result of the local mass effect of the GIC. Depending on the origin of the cyst, other complications include infection, haemorrhage or rupture of the cyst [3].

Diagnosis of a cyst or pseudocyst is difficult to ascertain pre-operatively as tissue biopsy or cytology can be difficult to attain. Ultrasound or CT scans done pre-operatively are a useful guide if the cyst can be seen to be arising from or located within an organ [2]. However, unless the cystic lesion is clearly seen to be arising from an organ, it is difficult to ascertain the origin as it may be abutting multiple organs due to the size. FNA of the cyst wall will be difficult unless there is a particularly thickened segment of the cyst wall. FNA of the cystic contents often prove to be of little yield. Furthermore, these can lead to infections or spillage of the contents [4]. As mentioned, serological tumour markers are non-specific and would not alter the course of management [5].

Abdominal cystic lesions can be broadly categorized as cysts or pseudocysts. Large cysts are uncommon and are often ovarian (functional, dermoid, cystadenomas), mesenteric (simple cysts, lymphangiomas) or peritoneal (mesothelioma, paraovarian) in origin and can be benign or malignant [6]. Mesenteric cysts are rare but tend to originate from the small bowel mesentery when it occurs [7]. Urachal cysts are located extraperitoneally. Pseudocysts are not uncommon but are often associated with pre-existing factors such as pancreatitis, ventriculoperitoneal shunts or surgical mesh placement. In this case, the most likely pre-operative diagnosis was that of a mesenteric cyst as this was a male patient with no predisposing history.

Surgical excision of the cyst is sufficient for treatment. However, dissection of large cysts can prove difficult especially in the deep posterior surface where the view is limited and retraction limited due to the size and weight. In this case, the author encountered difficulty freeing adhesions between the cyst and the intestines safely. In order to aid visualization, the cyst was decompressed under control. This drastically improved the surgical view and allowed the cyst to be easily retracted for adhesiolysis.

## 4 Conclusion

All patients with GIC should be offered surgical excision in view of the mass effect. Surgical excision alone is sufficient and will allow for resolution of the symptoms. The cystic lesion should be sent for histology for definite diagnosis.

**Table:** 

Learning points • Giant abdominal cystic lesions can be cysts or pseudocysts (see Chart 1). • Pre-operative imaging is essential to aid diagnosis and surgical planning. • FNAC is often not helpful. • Surgical excision of the lesion alone is often sufficient treatment. • Consider a controlled decompression to allow for retraction and visualization of the deep posterior surface.

## Funding

The author declares there was no funding involved.

## Ethical approval

No ethical approval was required as the author’s institution does not require approval for case reports of less than three patients. Consent was also obtained from the patient and the patient is not identifiable from the case report.

## Consent

Written informed consent was obtained from the patient for publication of this case report and accompanying images. A copy of the written consent is available for review by the Editor-in-Chief of this journal on request.

## Author contribution

Nern Hoong Kao was involved in all stages of the article.

## Registration of research studies

N/A.

## Guarantor

Nern Hoong Kao.

## Provenance and peer review

Editorially reviewed, not externally peer-reviewed.

## Declaration of Competing Interest

The author declares no conflict of interest.
